# Zeolite-Enhanced Portland Cement: Solution for Durable Wellbore-Sealing Materials

**DOI:** 10.3390/ma16010030

**Published:** 2022-12-21

**Authors:** Sai Vamsi Krishna Vissa, Cody Massion, Yunxing Lu, Andrew Bunger, Mileva Radonjic

**Affiliations:** 1 420 Engineering North, Petroleum Engineering Program, School of Chemical Engineering, Oklahoma State University, EN 420, Stillwater, OK 74074, USA; 2 Civil and Environmental Engineering, University of Pittsburgh, 3700 O’Hara Street, Pittsburgh, PA 15261, USA

**Keywords:** wellbore cement, zeolites, plugging and abandonment, self-healing barrier materials

## Abstract

Wellbore-plugging materials are threatened by challenging plugging and abandonment (P&A) conditions. Hence, the integrity and resilience of these materials and their ability to provide sufficient zonal isolation in the long-term are unknown. The present work focuses on investigating the potential to use zeolites as novel additives to the commonly used Class-H cement. Using four different zeolite–cement mixtures (0%, 5%, 15% and 30%, by weight of cement) where samples were cast as cylinders and cured at 90 °C and 95% relative humidity, the unconfined compressive strength (UCS) testing showed a 41% increase with the 5% ferrierite addition to the Class-H cement in comparison to neat Class-H cement. For triaxial compression tests at 90 °C, the highest strength achieved by the 5% ferrierite-added formulations was 68.8 MPa in comparison to 62.9 MPa for the neat Class-H cement. The 5% ferrierite formulation also showed the lowest permeability, 13.54 μD, which is in comparison to 49.53 μD for the neat Class-H cement. The overall results show that the 5% ferrierite addition is the most effective at improving the mechanical and petrophysical properties based on a water/cement ratio of 0.38 when tested after 28 days of curing in 95% relative humidity and 90 °C. Our results not only demonstrate that zeolite is a promising cement additive that could improve the long-term strength and petrophysical properties of cement formulations, but also provide a proposed optimal formulation that could be next utilized in a field trial.

## 1. Introduction

Over a period of a few decades, a multitude of oil and gas wells have been constructed to meet the growing energy demands of the world. However, once the oil and gas reserves are depleted and the wells reach a stage where they are not economically viable for production, they need to be plugged and abandoned (P&A) to avoid the leakage of residual hydrocarbons into the environment. According to the U.S. Environmental Protection Agency (EPA), around 281 kilotons of methane has been released into the atmosphere from abandoned oil and gas wells as of 2018 [1]. From a global warming perspective, it is of utmost importance to curb the leakage of these hydrocarbons into the environment. The United States Energy Information Administration (EIA) estimated the number of producing wells to have been reduced to 969,140 as of 2019, as compared to the 1,029,000 producing wells in 2014 [2]. An estimated 3000 wells and 650 platforms need to be P&A in the Gulf of Mexico alone, according to the Bureau of Ocean Energy Management (BOEM). There are roughly 29 million oil and gas wells that are orphaned all around the world, and a majority of them need to be P&A [1].

**Figure 1:**
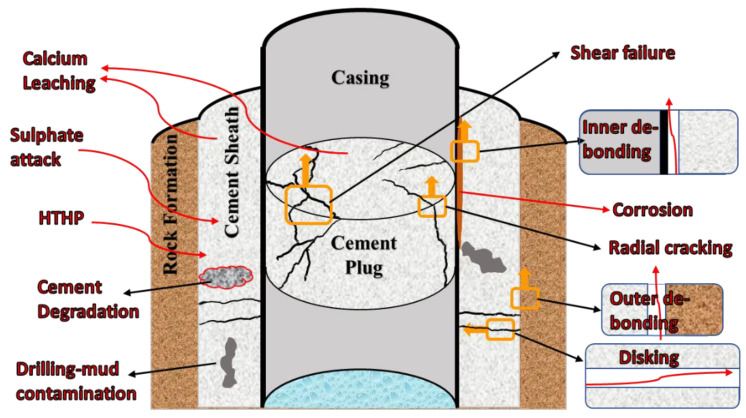
The various possible leakage pathways that could be observed in a wellbore cement plug due to the challenging P&A conditions [9].

The P&A of offshore wellbores creates several challenges, especially because of the adverse conditions encountered at the subsurface, with typical depths reaching over 2500–5000 ft below sea level. Usually, these wells are subjected to high-pressure and high-temperature (HPHT) conditions, and they are often also exposed to harsh chemical conditions [3,4,5]. Hence, this is an environment that poses a threat to the commonly used plugging material, i.e., Class-H/G cement, as it can cause the cement to fail, creating multiple potential leakage paths (Figure 1). For example, the inner debonding is created by the debonding that occurs between the cement sheath/plug and the casing, and the outer debonding occurs between the cement sheath and the rock formation of the reservoir into which the well was drilled. Disking fractures perpendicular to the well hole can occur in the cement sheath around the casing. Shear failure and radial cracking can take place in the cement plug, causing leakage pathways for hydrocarbons [6]. Fluids already residing within the subsurface wellbore, such as CO_2_, can also cause degradation and fracturing of the cement, which leads to more leakage [7]. Alternatively, the failure mechanism may already be present before the cement is placed, such as due to the failure to clean the wellbore of drilling mud, which results in contamination and weakening of the cement [8].

For several years, many plugging and abandonment protocols were followed and a variety of goals were proposed to ensure a robust seal of these abandoned wellbores [10]. Perhaps the most important overarching goal is caprock restoration. In nature, reservoir caprocks have been sustained in these harsh conditions for millions of years and, therefore, they act as a benchmark for the cement formulations and hydraulic and mechanical properties used for P&A. This research aims to mimic the properties of caprock and make the cement formulations as resilient as natural caprock.

The cement properties are affected by the early and late hydration of the cement paste, and the hydration characteristics can be controlled by adding various mineral and chemical supplementary materials [11,12,13,14]. After hydration, cement pastes are typically brittle in nature. For surface applications, this problem is overcome by using reinforced cement concrete with steel rebar, and by adding coarse and fine aggregates to the cement mixture. This cannot be used for P&A, on the other hand, due to challenges such as segregation [15], as offshore wellbore construction and P&A operations require this cement to be pumped over very long distances, sometimes thousands of feet downhole. Hence, cement is pumped as a paste for wellbore construction operations. Keeping the cement stable all through the pumping process, the hydration process and after the fact demands highly advanced solutions. Nano- and micro-additive materials [16,17,18,19] and chemical retarders are added to the cement slurry before pumping to achieve the desired rheological properties. This addition could also enhance hydration and, eventually, improve various cement properties, providing durability over the long run. Despite the many insights acquired from concrete [20,21,22,23,24] used for construction on land, and the major similarity in its cement chemistry with offshore wells, special care needs to be taken in the case of offshore wells [25,26,27,28,29,30]. 

The cement formulations used for P&A need to be resistant to the harsh subsurface conditions to avoid any hydrocarbon leakage and ensure zonal isolation [4]. The durability of the cement formulations is the biggest concern for long-term sealing of wellbores. The aim of the formulation design is to have optimal petrophysical properties to ensure the integrity of the wellbore, to prevent leakage of hydrocarbons, and to have higher resistance to HPHT conditions and harsh downhole chemical conditions (i.e., the presence of corrosive/acidic conditions, which have a deteriorating effect on cement [31,32]). The cement also needs to have more strength and resilience to avoid failures, which encompasses the elastic over-brittle nature of hydrated cement pastes, and also needs to have the ability to self-heal in case of a failure [33,34].

**Figure 2:**
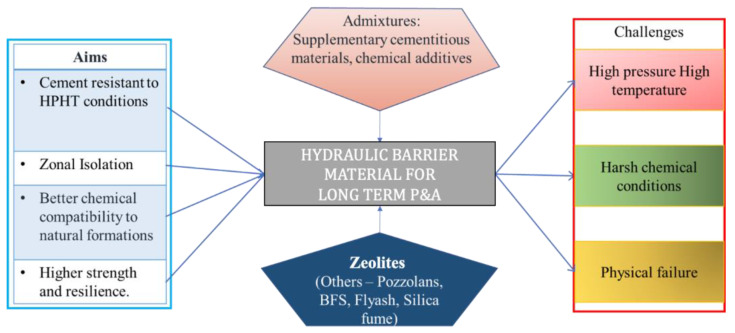
Challenges in usage of cement for subsurface conditions, additives and the aims of P&A.

Therefore, a multitude of challenges are clear, as summarized in Figure 2 (left column). In order to tackle these challenges to achieve the P&A goals, several cement additives have been studied in the past and present [35,36] to improve the quality of cement formulations. However, zeolites are a relatively new class of additive materials that have received very little research attention for the specific application of P&A. Zeolites are a class of aluminosilicate minerals with a characteristic framework structure. Many can be found in abundance in nature. Previous research has shown that by adding zeolite into cement, the mechanical, hydraulic and geochemical properties can be impacted [37,38,39,40,41]. The addition of a specific zeolite (ferrierite) was also found to show self-healing properties in the case of geothermal cement formulations [34]. Ferrierite (FER) is a group of zeolite minerals that consists of three similar species, [42] namely:

- Ferrierite-Mg ([Mg_2_(K,Na)_2_Ca_0.5_](Si_29_Al_7_)O_72.18_H_2_O);
- Ferrierite-Na ((Na,K)_5_(Si_31_Al_5_)O_72.18_H_2_O);
- Ferrierite-K ((K,Na)_5_(Si_31_Al_5_)O_72.18_H_2_O).

The successful application of FER in the geothermal industry [43] results in self-healing, and in the enhanced mechanical and hydraulic properties of FER cement. Thus, the objective of the study reported in this paper is to explore the feasibility of transferring the techniques of FER cement to be applied to P&A to leverage the challenging conditions and achieve the goals of caprock restoration and a more resilient P&A system. In the present work, the Class-H cement formulations with various percentage additions of FER (5%, 15% and 30%) were prepared and cured under representative downhole conditions. Experiments were carried out to evaluate the effects of FER on the microstructural properties of cement, and to obtain engineering parameters such as Young’s modulus, axial strength, and hardness. The pore-scale measurements and microanalysis were carried out to investigate the micromechanical origins of the observed impacts of FER on cement properties and the fundamental scientific mechanisms behind it.

## 2. Experimental Methods and Material Characterizations

### 2.1. Materials and Methods

Class-H cement used for this study was obtained from Halliburton. The zeolite rock used for the studies was obtained from Lovelock, NV, USA, and was provided by the Trabits Group. The mineralization was hosted in the bedded tuff in the rock formation. The zeolite cement used as a reference in our studies (its chemical composition is shown in Table 1) was also obtained from the Trabits Group and was produced at the Maryneal, TX, USA plant of the Buzzi Unicem company. A CFR-3 friction reducer and a D-Air 5000 defoamer were also used in the final formulation and were obtained from Halliburton. Bentonite was another material used as a densifier to make the final formulation have a density of 16.4 pounds per gallon (ppg). The zeolite rock was ground into fine powder before being added to cement by using a Spex ball mill. Powdered zeolite particles were of an approximately 30–90 μm size range and the maximum particle size was ensured by sieving the powdered zeolite through a 90 μm mesh. The SEM, EDS and XRD studies performed in earlier works established the fact that zeolites act as scaffolding material for cement hydration, given the fact that they remain in a stable phase in the hydrated cement cores. The stability of chemically treated zeolite in hydration conditions and subsurface chemical and temperature conditions was observed. With these methods, a comparative study of the cement properties is conducted for the cement formulations with varying zeolite content, including observations of the interaction between cement and zeolite.

### 2.2. Cement Slurry Preparation

American Petroleum Institute (API) Standard 10 B for wellbore cementing was followed to prepare the cement slurries [44]. The cement slurries were prepared by, firstly, taking the Class-H cement powder (<120 μm), respective additives (CFR-3, bentonite, and D-air-5000) and micronized zeolite (~30–90 μm) powder in the respective percentages by weight of cement (bwoc) and hand mixing them in a container. Then, this hand-mixed dry cement mixture with all the additives was gradually transferred into an industrial blender in a span of 15 s, which was ran at a slower speed (~4000 rpm) and contained the calculated amount of water for the slurry. The blender was then turned on to high speed (~12,000 rpm) once the lid of the blender was secured. This should be run for 35 s to ensure that there are no lumps that are formed in the slurry. The summary of these formulations is given in Table 2.

The slurry was then transferred to casting molds with the dimensions that are prescribed for various tests. Sample cores for triaxial testing were prepared in molds that had a 1.18-inch diameter and 2.4-inch length, whereas cores with a 1-inch diameter and 2-inch length were used for all other testing purposes including UCS and petrophysical studies, and for preparing pucks for SEM, EDS, Raman spectroscopy and indentation. The respective cores were demolded after 24 h, submerged in a Ca(OH)_2_ solution and cured in an ESPEC 12-4NAL humidity- and temperature-controlled environmental chamber. They were placed at 90 °C and a 95% relative humidity (RH) to simulate the downhole hydration conditions and cured for 28 days. Some cores of each formulation were cut into pucks and polished using an Allied Rancho Dominguez, CA, USA MultiPrep™ polisher to a ~0.6 μm uniformity for various characterization techniques. 

The Class-H cement used in these designs is the API-recommended cement for P&A applications, especially for use under the HPHT conditions observed in the Gulf of Mexico. The amounts of defoamer (D-air 5000), friction reducer (CFR 3) and densifier (bentonite) to be added to the cement design were calculated in such a way that the final density of the resultant cement design for each particular percentage of ferrierite added was 16.4 ppg.

### 2.3. Raman Spectroscopy

Raman spectroscopy was also performed on these pucks for each of the formulations using a Oxford Instruments WiTec Concord, MA, USA alpha300 R tabletop Raman microscope equipped with 532 nm green and 785 nm red lasers. The polished pucks were placed under the microscope and large area scans and profilometry were performed using the 532 nm green laser at ~5 W laser power, which collected 30 accumulations per scan with a 1.0 s integration time for point scans. A 0.1 s integration time, 100 points per line and 100 lines were used for large area scans and profilometry covering a 1000 × 1000 μm area on the polished surface.

### 2.4. X-ray Fluorescence (XRF)

The unhydrated Class-H cement used in the cement formulations was studied and compared with commercial zeolite cement and plain zeolite by using XRF to obtain the various oxide contents in these samples to indicate how the replacement of cement with zeolite varies from plain Class-H cement. These tests were carried out using an EDAX Ametek Mahwah, NJ, USA Orbis Micro-XRF microscope with samples placed inside of a plastic cylinder with an approximately 1-inch diameter and 0.5-inch height that were filled and loaded in the testing chamber. Test settings of 50 kV, 1000 μA and 5 iterations were used.

### 2.5. X-ray Computed Tomography (CT)

CT scanning of the cores was conducted after triaxial testing to show the fracture network within the different FER-added cores. This was carried out using the North Star Imaging Rogers, MN, USA M-5000 industrial Computed Tomography (CT) scanner at the National Energy Technology Laboratory (NETL). The scans were reconstructed through the North Star Imaging Rogers, MN, USA efX-CT^®^ software and the resultant 3D images had a voxel resolution of (32.9 mm)^3^. Image segmentation of the open voids and fractures from the cement matrix was performed using pixel segmentation with ilastik [45]. Further postprocessing of the images and visualization were performed using ImageJ/FIJI [46].

### 2.6. Microindentation

Indentation tests were performed on the pucks of neat Class-H cement and 5%, 15% and 30% FER-added Class-H cement formulations using a Nanovea Irvine, CA, USA PB1000 hardness tester. The indents were later studied under SEM for further examination. For this testing, the instrument was calibrated using a micro-Vickers diamond tip that has a 1140 GPa elastic (Young’s) modulus (E*_i_*) and a Poisson’s ratio (v*_i_*) of 0.07, following ASTM Standard E2546, and uses a finely polished stainless-steel puck with known properties, which was the standard calibration sample [47]. For this experiment, 25 indentations were taken per sample in a 5 × 5 matrix configuration, each with a spacing of 400 μm and tested at a maximum load of 5 N, a loading and unloading rate of 10 N/min, and a creep time of 300s. The load vs. depth was then plotted and analyzed using ASTM E2546 with 10–50% of the unloading curve and a cement Poisson’s ratio approximated to be 0.3.

### 2.7. Unconfined Compressive Strength (UCS)

UCS testing is widely used for characterizing cement strength [48,49]. A servo-hydraulic computer-controlled load frame from James Cox and Sons Colfax, CA, USA was used to perform this testing. The UCS was measured following the ASTM standard procedure. This entails preparing the samples at a 1:2 aspect ratio and grinding the top and bottom faces to be parallel to within 0.001 of an inch. Samples were loaded at a constant strain rate of 1 × 10^−5^/s. The load was applied in the following manner:

(1) The piston was loaded to be actuated under stress control until 50 psi was reached.
(2) The piston was then switched to be under strain (position) control.
(3) The sample was loaded at a constant rate until failure, and then unloaded.

The peak stress condition was calculated from the load cell force measurements and sample diameter.

### 2.8. Triaxial Tests at Elevated Temperature

To obtain a better assessment of the strength properties under downhole conditions, triaxial compression tests were performed at an elevated temperature. A temperature-controlled, Hoek-type triaxial compression cell (Roctest Ltd., Saint-Lambert, QC, Canada) was used for these tests. The triaxial cell consists of three main parts: the axial deviatoric loading system, the confining pressure system, and a temperature controller. In this test, downhole conditions were simulated by creating confining stress and elevated temperatures. Deviatoric stress controlled by the INSTRON-600DX system (max load of 600 KN) was applied in a gradually increasing manner until the specimen failed, while the confining pressure and temperature were also applied. A high-pressure syringe pump was used to maintain the desired confining stress. In the experiments performed for this work, a confining pressure of 13.7 MPa was applied, allowing the precise measurement of the volume change in the specimen associated with it. A controlled temperature of 90 °C was provided by wrapping the Hoek cell with heating tape. After the desired system temperature was achieved and stabilized, the confining pressure and vertical load were increased to the targeted downhole pressure value, and thus, the specimen was initially loaded isotropically, and then the deviatoric load was increased until the specimen failed. Following ASTM-D7012, the specimen was tested at a constant rate ($3.3\times 10^{-6}$ m/s) so that the specimen would fail in approximately 10 to 15 min [50,51]. During the test, the load frame recorded the axial position of the top piston. These data were used to derive the axial strain ($e_{A}$) of the specimen.

### 2.9. Petrophysical Properties (Porosity and Permeability)

The petrophysical properties of these sample cores were measured and compared to determine the best formulation. The porosity and permeability of these samples need to be negligible in order to maintain a proper geological seal, and hence, it is important to know these properties. The porosity of the samples was measured using the Core Lab Tulsa, OK, USA UltraPore 300 Helium Porosimeter and the permeability was measured using the Core Lab Tulsa, OK, USA Nano-Perm Permeameter. 

Mercury intrusion porosimetry (MIP) was also carried out for the best performer among the formulations to obtain an understanding of the pore structure and distribution. Samples were immersed in mercury in a pressure-sealed chamber, and the pressure of the surrounding mercury was gradually increased from 5 up to 55,000 psia (0.03 to 379.21 MPa). The relationship between the injection pressure and mercury saturation was used to calculate the parameters of porosity and pore throat size distribution.

## 3. Results

### 3.1. Microstructural Characterisation

#### 3.1.1. Chemical Characterization I (Raman Spectroscopy)

**Figure 3:**
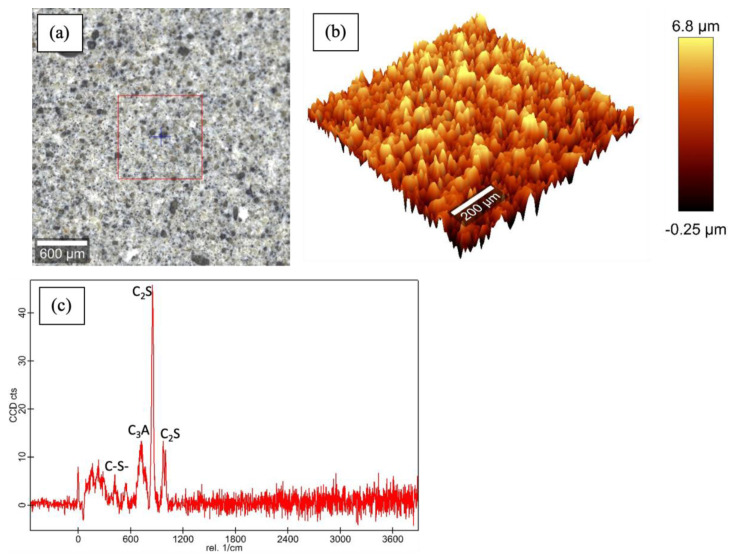
Raman results for polished cores of neat Class-H cement: (**a**) optical micrograph of polished cement core; (**b**) large-area profilometry scan of the cement; (**c**) phase peaks at 600–630 nm (C-S-H), 740 nm (C_3_A), 842 nm and 972 nm (C_2_S) were observed.

**Figure 4:**
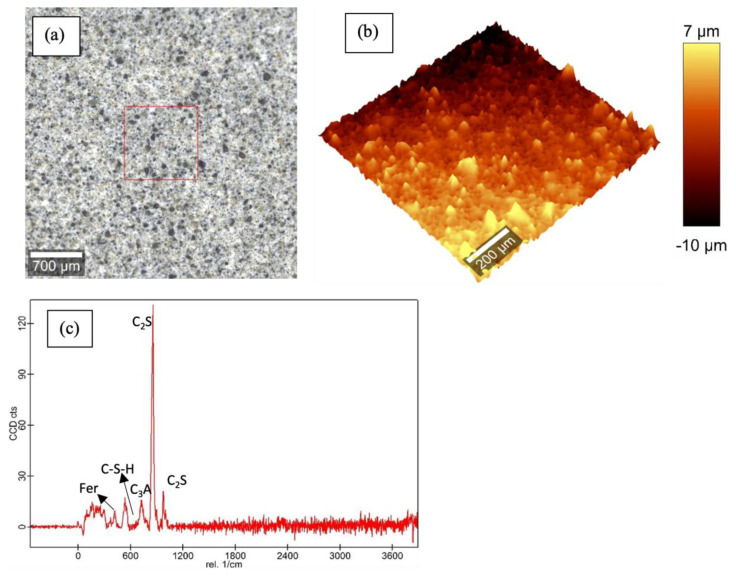
Raman results for polished cores of 5% ferrierite-added Class-H cement: (**a**) optical micrograph of polished cement core; (**b**) large-area profilometry scan of the cement; (**c**) phase peak at 416 nm (ferrierite (Fer)). C-S-H, C_3_A and C_2_S are the same as those mentioned in the plot for neat Class-H cement.

**Figure 5:**
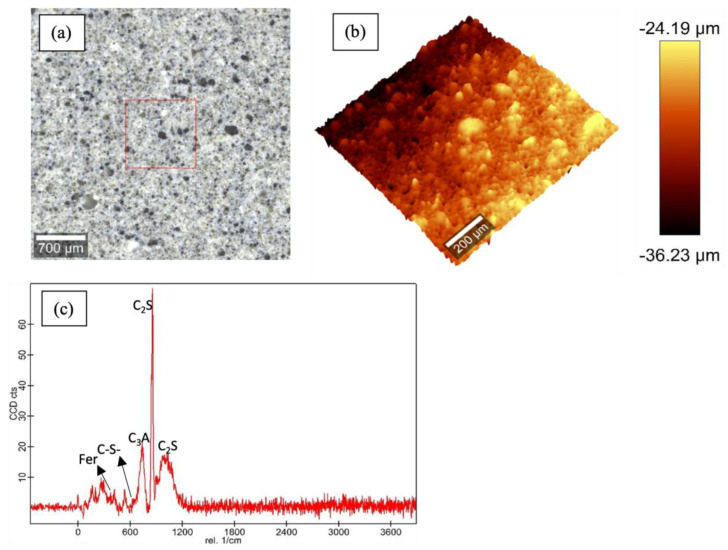
Raman results for polished cores of 15% ferrierite-added Class-H cement: (**a**) optical micrograph of polished cement core; (**b**) large-area profilometry scan of the cement; (**c**) phase peaks identical to those observed in Figure 3 for ferrierite, C-S-H, C_3_A and C_2_S.

**Figure 6:**
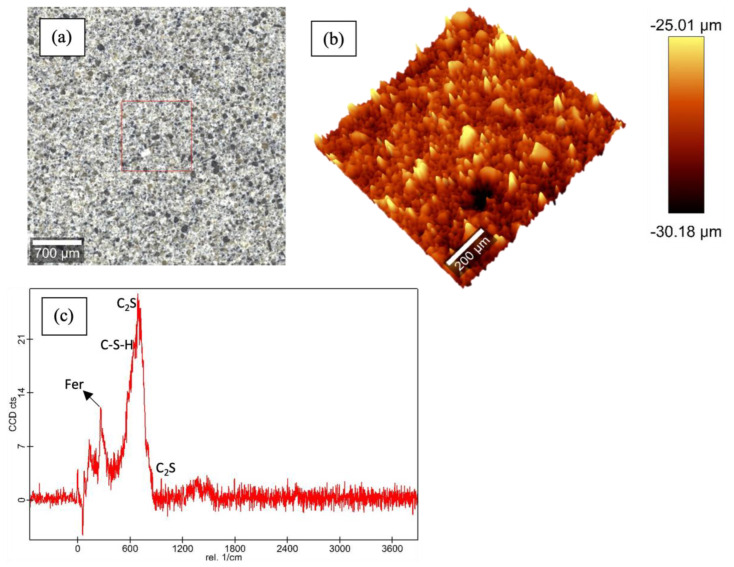
Raman results for polished cores of 30% ferrierite-added Class-H cement: (**a**) optical micrograph of polished cement core; (**b**) large-area profilometry scan of the cement; (**c**) phase peaks for ferrierite, C-S-H and C_2_S are observed in this plot. However, C_3_A was not resolved well in these scans.

Figure 3, Figure 4, Figure 5 and Figure 6 show the Raman data for 5%, 15% and 30% ferrierite-added Class-H cement samples compared to the neat Class-H sample. These visualizations show how characteristic ferrierite phases are observed in all the ferrierite-added samples, which further supports the hypothesis. It also includes a large-area Raman scan that shows the phase distribution and a profilometry image of the phase morphology.

#### 3.1.2. Chemical Characterization II (X-ray Fluorescence (XRF))

**Figure 7:**
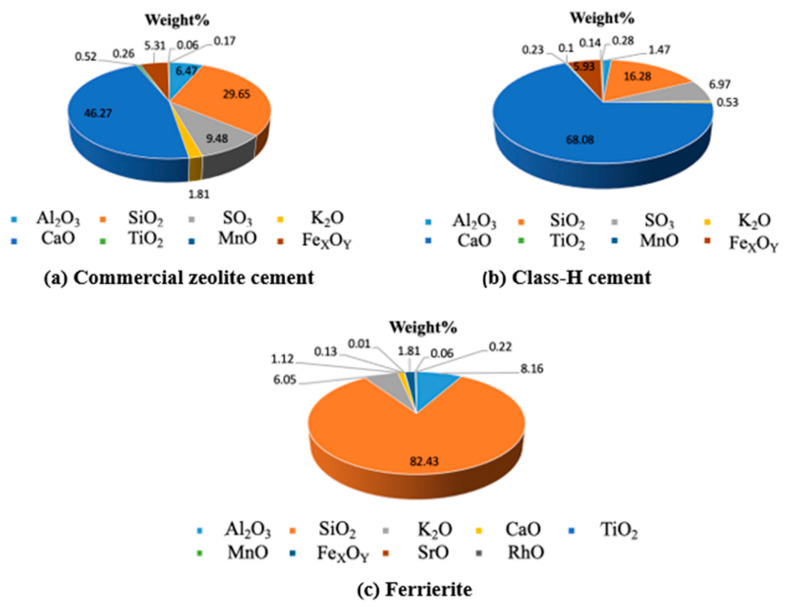
XRF results showing the percentage distribution of various oxides present in unhydrated (**a**) commercial zeolite cement and (**b**) plain Class-H cement; (**c**) XRF data plot for ferrierite, which is a zeolite. The similarity in phases and or oxide compositions indicates that there are no new/intermediary phases that are formed. However, the addition of zeolite reduces the CaO content, which would eventually result in the more brittle phases of cement with hydration.

The XRF results are shown in Figure 7, exhibiting the distribution of various oxides present in the cement. This is a nondestructive technique, as mentioned in the Experimental Methods and Material Characterizations. The distribution of the chemical oxides in the material is shown. The charts below show the comparison between the unhydrated Class-H cement and unhydrated zeolite cement (commercial geothermal zeolite cement) that highlights the presence of a stable zeolite phase, which can be noted in the comparison between the phases of pure ferrierite and commercial geothermal zeolite cement. 

### 3.2. Three-Dimensional Internal Morphology Characterization (CT)

**Figure 8:**
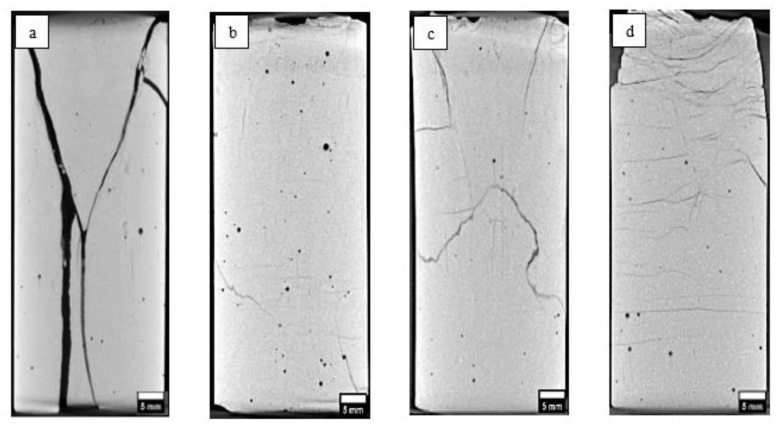
CT scans of cement cores after failure under triaxial testing at a confining pressure of 13.7 MPa and 90 °C: (**a**) neat Class-H cement showing the failure in comparison to the others; (**b**) 5% ferrierite-added cement; (**c**) 15% ferrierite-added cement; (**d**) 30% ferrierite-added cement.

The internal fracture morphology of the ferrierite-added cement samples was studied using computed tomography (CT) after triaxial testing (Figure 8). Based on the apparent width and persistence of the generated cracks, the largest cracks occurred in neat cement. Additionally, the 5% ferrierite case shows the best performance in terms of minimizing fracture density, aperture and persistence. 

### 3.3. Micromechanical Characterization (Microindentation)

**Figure 9:**
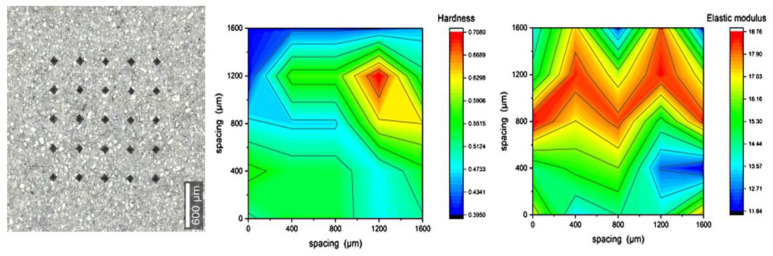
Indentation results for polished cores of neat cement with 25 indents, represented as maps of hardness (avg. of 0.506 GPa) and elastic modulus (avg. of 15.72 GPa).

**Figure 10:**
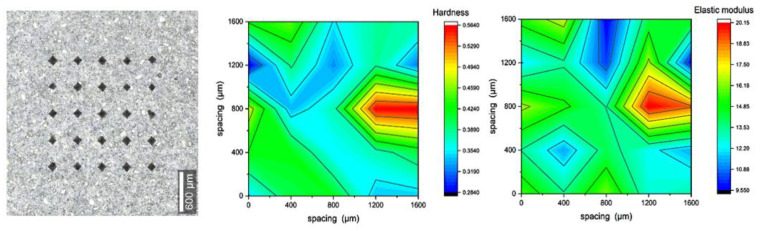
Indentation results for polished cores of 5% ferrierite-added Class-H cement with 25 indents, represented as maps of hardness (avg. of 0.394 GPa) and elastic modulus (avg. of 14.03 GPa).

**Figure 11:**
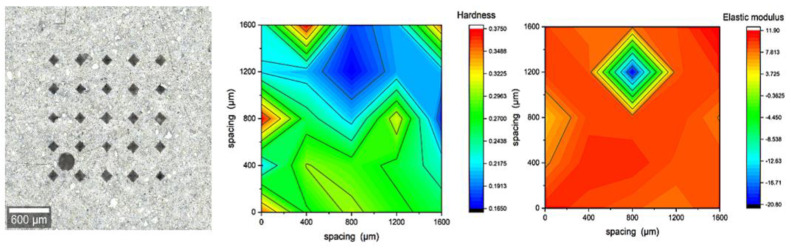
Indentation results for polished cores of 15% ferrierite-added Class-H cement with 25 indents, represented as maps of hardness (avg. of 0.319 GPa) and elastic modulus (avg. of 11.01 GPa).

**Figure 12:**
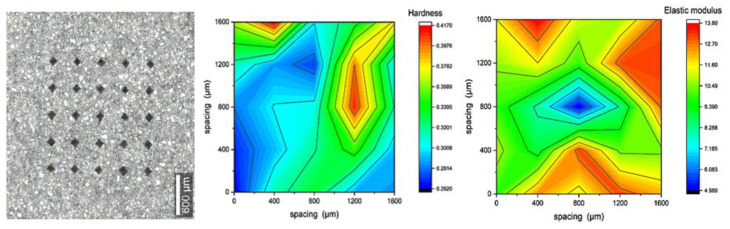
Indentation results for polished cores of 30% FER-added Class-H cement with 25 indents, represented as maps of hardness (avg. of 0.256 GPa) and elastic modulus (avg. of 7.88 GPa).

**Figure 13:**
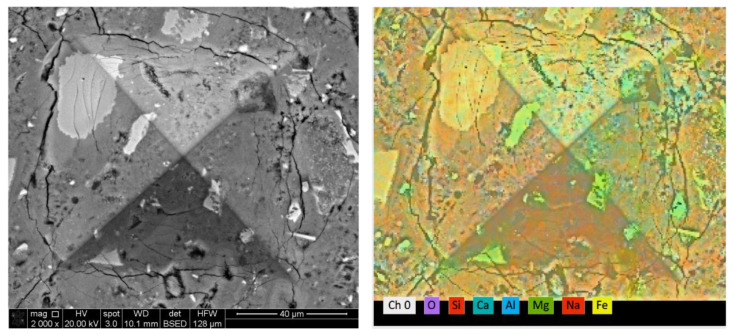
An EDS map corresponding to an SEM image showing a single indent on neat cement. Indentation was conducted for the other formulations (for 5%, 15% and 30% ferrierite-added Class-H cement) as well, and SEM and EDS studies were undertaken to see if there is any noticeable surface chemical change upon failure.

The results shown in Figure 9, Figure 10, Figure 11 and Figure 12 are of 5 × 5 indentation grids on a highly polished cement surface, for which the obtained hardness and elastic modulus are mapped adjacently. These results were used to calculate the average hardness and elastic modulus of the sample. The hardness and elastic modulus were calculated from the force vs. displacement curves obtained from each indent by using the software interface of the indenter. The samples were further processed by mapping the hardness and elastic modulus corresponding to each indent and representing them as mentioned above. These indent marks were also observed under SEM to obtain the morphology and the chemical characterization using EDS to show the phase stability of cement phases under stress, as shown in Figure 13. This test would be more useful for observing self-healing after the failed cores are kept in the environmental chamber again at high-temperature and high-humidity conditions, such as the ones used for the hydration and curing of the cement cores. Self-healing would possibly occur due to secondary hydration and the corresponding phase changes would be visible in the EDS maps acquired for the indenter marks post-curing.

Future work is planned to test the indentation marks in a more detailed manner and to correlate them with the EDS studies to see if there is any chemical change in the matrix. This could help us better characterize secondary hydration, which would be important for understanding the potential for the self-healing of damage sustained by the materials.

### 3.4. Mechanical Strength Characterisation

#### 3.4.1. Mechanical Property Characterization I (Unconfined Compressive Strength (UCS))

**Figure 14:**
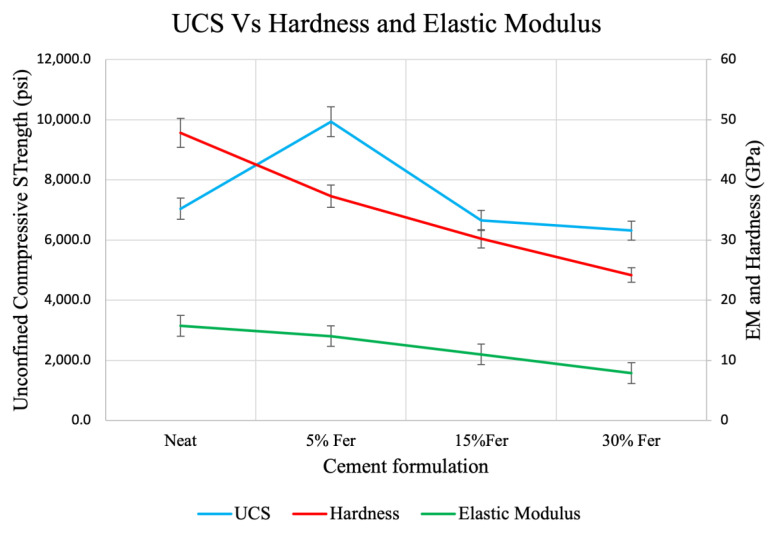
UCS versus indentation-derived hardness and elastic modulus for 5%, 15% and 30% ferrierite-added Class-H and neat Class-H cement, showing slightly similar trends. UCS is the highest for 5% ferrierite-added cement at 9934.3 psi compared to neat UCS at 7041.5 psi. Elastic modulus and hardness, on the other hand, are highest for neat Class-H with averages of 15.72 GPa (EM) and 47.82 (hardness) followed by the 5% ferrierite addition at 14.03 GPa (EM) and 37.3 (hardness). The hardness and elastic modulus decrease gradually with the increase in the percentage addition of ferrierite, which is a similar trend as that of UCS.

The addition of 5%, 15% and 30% ferrierite to Class-H cement impacts the mechanical properties in a manner illustrated in Figure 14. Here, the impact on the UCS is compared to the hardness and elastic modulus, where the latter two properties were observed through the indentation testing. Although the elastic modulus and hardness follow a similar trend, with the 5% ferrierite having a smaller magnitude compared to the neat Class-H cement, the UCS results show the highest values for the 5% ferrierite-added Class-H cement cores at 9934 psi compared to 7041 psi for the neat Class-H cement. Due to this, the 5% ferrierite addition is initially chosen as the best performing formulation. This trend is also evident in other mechanical characterization techniques such as the triaxial test, which is included in the next section.

#### 3.4.2. Strength Property Characterization II (Triaxial Strength Testing)

**Figure 15:**
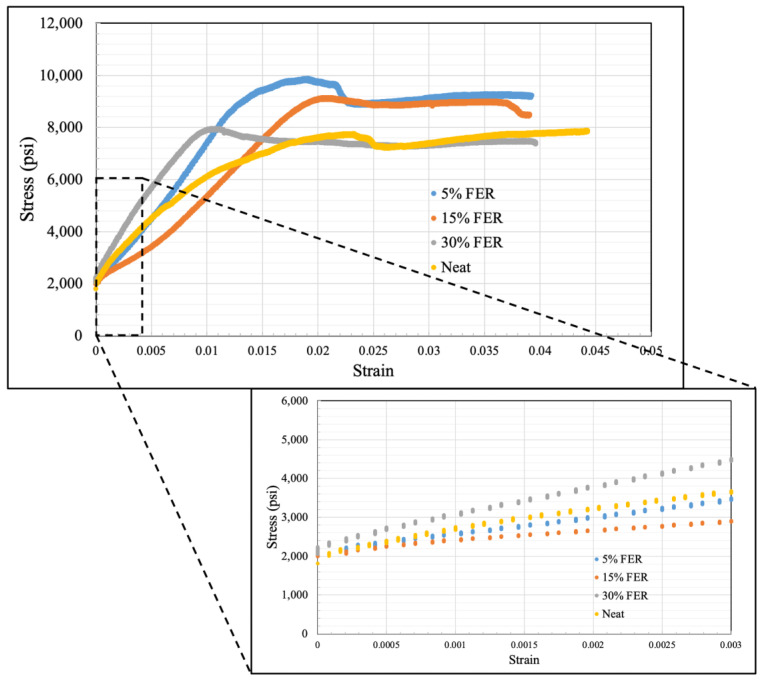
Triaxial test results for 5%, 15% and 30% FER-added and neat Class-H cement at 13.7 MPa confining pressure and 90 °C; 5% FER-added cement has the highest axial stress at 68.8 MPa, followed by 62.9 MPa and 54.5 MPa for the 15% and 30% additions, respectively, whereas the neat Class-H cement sample has a maximum of 53.3 MPa. These results coincide with the UCS results showing a similar trend, with 5% having the best performance. The initial linear regime is shown in a magnified scale in the inset, and the slope of this initial linear regime is used to calculate Young’s modulus.

Triaxial testing was carried out under confinement and elevated temperatures, with the intent of considering stresses and temperatures that are relevant to typical downhole conditions. Four sample cores with 5%, 15% and 30% ferrierite bwoc additions to the Class-H and neat Class-H cement slurry designs were tested at the same confining stress (13.7 MPa) and temperature (90 °C) conditions. The strain-stress curve for each specimen was obtained, as shown in Figure 15. The initial linear portion of this curve was used to obtain Young’s modulus. These results show that the 5% FER sample has the highest axial peak stress of 68.8 MPa (which is the confined compressive strength for this confinement level). Additionally, the axial peak stress monotonically decreases when the FER percentage increases. However, the 30% FER sample, which has the lowest axial peak stress out of the other two doses, still shows higher axial stress than the neat cement. Despite that, the Young’s modulus of neat cement is higher than the 5% FER and 15% FER samples, but lower than the 30% FER sample. 

### 3.5. Petrophysical Property Testing

Once the strength properties are tested, the last characterization that is required to determine the suitability of the material for plugging and abandonment purposes is the testing of the petrophysical property. Zonal isolation is the most important factor for a material to be considered as a P&A solution, and hence, it is important to characterize the porosity and permeability of the cement. 

**Figure 16:**
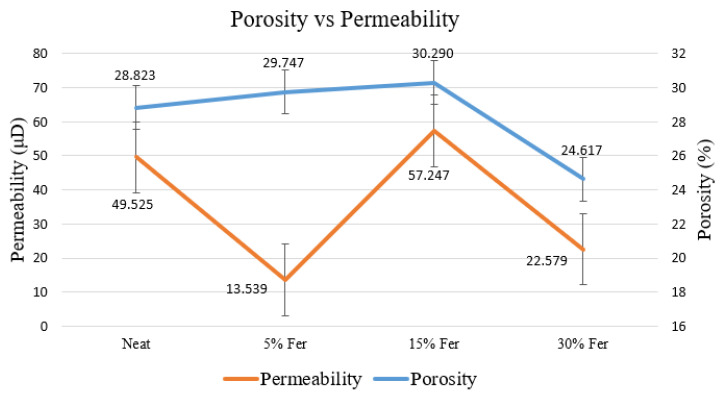
Average porosity and permeability values of 5%, 15% and 30% FER-added and neat Class-H cement measured using Core Lab UltraPore 300 Porosimeter and Nan-Perm Permeameter. Although 30% ferrierite has the least porosity due to better hydration, the 5% ferrierite addition shows the best zonal isolation as it has the least permeability of 13.54 μD in comparison to neat Class-H cement (49.53 μD).

Porosity is the percentage of the sample core that is filled with pore spaces or void spaces. This also has an impact on the material integrity, as pore spaces could become initiation points for a fracture to begin under stress. This is measured via a porosimeter by taking a cement sample core in a matrix cup. The porosity is calculated based on the grain volume obtained from the combined gas law equation (P_1_V_1_ = P_2_V_2_) where the gas pressure remains known, and the volume is calculated from the gas that expands into the pore spaces when a constant gas pressure is applied. Porosity needs not necessarily translate into permeability, as the pore spaces could be isolated. Hence, permeability also needs to be measured, as it indicates the ability of a material to allow the passage of liquids or gases through it. As observed in porosity measurements through helium gas expansion porosimetry, the addition of ferrierite slightly increases the porosity of the cement cores, but decreases permeability by 30%, which could be attributed to the enhanced hydration due to the stored pore water within the ferrierite mesopores. The permeability calculations were obtained using Darcy’s law, which is based on the flow rate of helium gas passed through a sample core at a constant pressure. This is conducted under a confining pressure to simulate the downhole conditions to obtain a wholistic measurement of a simulated cement plug. Figure 16 shows the porosity compared to the permeability values that were obtained for the 5%, 15% and 30% ferrierite-added cement samples in comparison to the neat Class-H cement.

**Figure 17:**
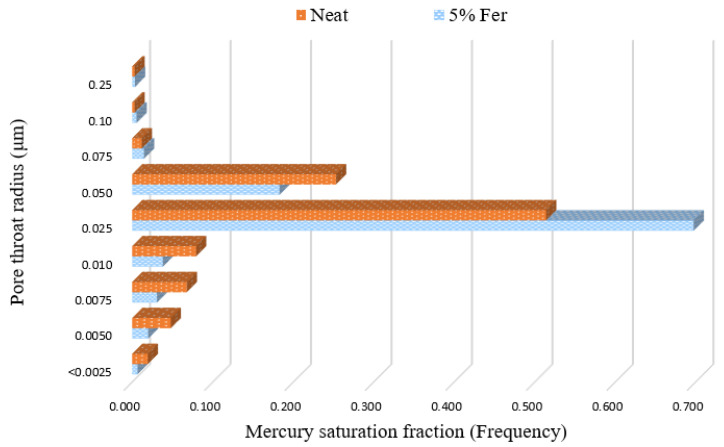
Pore-throat radius as measured via mercury intrusion porosimetry, where the greatest pore-throat radius distribution is in the nano-range (0.025 μm), supporting the hypothesis that an increase in porosity is attributed to the nano-porosity introduced by the addition of ferrierite. Ferrierite is a zeolite mineral that is characterized by its crystalline structure with a porosity in the nano-range, and hence, it could also support the fact that the porous crystalline structure of the zeolite is preserved in the cement matrix even after hydration.

Further testing to study the pore network characteristics was carried out via mercury intrusion porosimetry (MIP). This testing was conducted on neat and 5% ferrierite cement (due to its results in the other testing showing the greatest improved results) to correlate the results with the helium gas porosimetry measurements. Mercury intrusion porosimetry gives insight into the distribution of the range of pore-throat radii (Figure 17). Despite having a higher frequency of distribution in comparison to the neat cement, these pore-throat sizes are mostly distributed in the nanometer range, and hence, do not directly contribute to permeability. This can be noted in the permeability results. Hence, the addition of ferrierite does not negatively impact the properties that are important for the zonal isolation of the cement but may instead enhance them.

## 4. Discussion

Previous work [52] has well established that zeolites do not participate in Portland cement hydration reactions at moderate temperatures, except in simulated high-pressure and high-temperature downhole conditions, as reported by Pyatina et al., 2018 [34]. This study examines the potential enhancement of hydrated Portland cement with the addition of a zeolite ferrierite to cement slurries in percentages of 5, 15 and 30%. Tests were conducted with the above additions of ferrierite added to Class-H cement formulations, and the results were compared with the properties of neat Class-H cement. A chemical evaluation was conducted to test the initial hypothesis that ferrierite does not show pozzolanic reactivity at a 90 °C curing temperature, but rather acts as the scaffolding or reinforcement material without undergoing any chemical or structural change. Raman spectroscopy confirmed that the peaks of ferrierite is consistent in all the ferrierite-added cement cores at 416 nm, indicating the chemical resilience of this mineral within an ordinary Portland cement (OPC) in high-pH environments. The data show that the ferrierite existed as an unreacted filler in the hydrated cement matrix even after 28 days of curing at 90 °C and 95% RH. XRF was performed for the unhydrated commercial geothermal zeolite cement and plain Class-H cement powders. The results show that the ferrierite replacement in the cement reduces the CaO content responsible for the more reactive phases in cement, which tends to be subjected to Ca leaching in a longer run exposure to harsh, low-pH conditions. These results indicate that cement replacement by natural zeolites, such as ferrierite, could be advantageous for the cement designs needed for geothermal and CO_2_ storage projects, as well as for the permanent plugging and abandonment of wellbores and orphan wells. 

UCS data were obtained for neat, 5%, 15%, and 30% ferrierite-added cements, and in comparison to the neat Class-H cement formulations, the cores that had ferrierite added showed a clear increase in strength for all percentages. It was observed that the 5% ferrierite-added cement cores showed an average compressive strength of 68.49 MPa compared to neat cement cores with an average of 48.55. Even more significant are the results from the testing under triaxial conditions, where a similar trend was observed in that the confined compressive strength (CCS) of the 5% ferrierite addition showed the best performance, being the highest at 68.8 MPa (at 13.7 MPa confinement). This is significantly improved in comparison to the CCS of neat cement, measured here as 53.3 MPa. The accompanying Young’s modulus was found to be lower for 5% and 15% ferrierite-added cement formulations, indicating their less-brittle nature in comparison to neat cement. However, it was observed that the modulus was higher in the 30% ferrierite addition than in neat cement, indicating that the optimal addition of ferrierite should be somewhere in the range of 5–15% (in the absence of other additives). 

The microscale hardness testing was carried out using a microindenter to observe the matrix micromechanical properties of cement cores in order to support the bulk properties and their enhancement, as seen in the UCS and triaxial tests. The results from all the mechanical property tests that were performed are summarized in Table 3, as an average of the observed results from UCS, triaxial and indentation tests for all four cement compositions. The respective plots were presented earlier in the Results section. 

To obtain an understanding of the petrophysical properties, the ferrierite-added cement cores and neat cement cores were tested for porosity, permeability, and pore size distribution. Although helium gas porosimetry did not show a major change in porosity, with differences only ranging between 1–2%, an important observation was made using MIP. A majority of the pore-throat radii were in the nanometer range, which is favorable for effective zonal isolation in a plugging and abandonment scenario. On the other hand, permeability results for 5% and 30% ferrierite-added cement cores showed lower values of 13.5 μD and 22.58 μD, respectively, as compared to neat cement cores with a permeability of 49.53 μD, suggesting that zeolite can contribute to improving the sealing capacity of the cement, which is critical in the primary cementing of wellbores as well as in permanent plugging. Table 4 shows the unconfined compressive strength along with petrophysical properties of porosity and permeability.

## 5. Conclusions

The addition of FER to the Class-H cement slurry enhances the mechanical properties as consistently observed through UCS, triaxial and microindentation testing. The UCS results showed a 41% increase in the compressive strength for the 5% ferrierite-added cement formulation (68.5 MPa) compared to the neat cement formulation (48.55 MPa). Similar results were observed in triaxial testing, which showed a 29% increase in the confined compressive strength for the 5% ferrierite-added cement formulation (68.13 MPa) in comparison to neat cement (53.29 MPa). Indentation results indicated that the cement matrix mechanical properties exhibited a more ductile behavior for ferrierite-added cement formulations when compared to neat cement. Furthermore, the petrophysical properties had a notable enhancement, as observed in the porosity and permeability measurements, and the porosity was determined to shift toward the nanometer range when ferrierite was added. The permeability showed that 5% and 30% ferrierite additions had the lowest permeability in comparison to neat cement. At the same time, the micro-characteristics indicate that the zeolite ferrierite used in this study acts as scaffolding for the Portland cement hydration products, primarily due to it porous crystalline structure that provides a large surface area for the hydration to take place, including the potential for seed crystals. The stability of ferrierite in the hydrated cement matrix was established with the Raman spectrometry results, which are in line with the EDS and XRD results from our earlier work, showing that these zeolites display less reactivity by themselves, but are very active in hydration when enhancing the reaction with cement. These results not only demonstrate that the ferrierite could be a promising additive to cement that provides better zonal isolation, but they also identify a candidate for the optimal addition percentage range of the zeolite for P&A purposes. Specifically, the results indicate that the 5% ferrierite addition had the most significant enhancement with respect to both mechanical and petrophysical properties, making it the recommended formulation for further studies that could include a field trial using the ferrierite additive in an actual plugging and abandonment application.

Another direction of future work is the potential for zeolite to be used in combination with other additives that could improve cement regarding other failure modes. Similar work has shown that olivine added to wellbore cement can help to prevent deterioration under harsh conditions [53] and graphene nanoplatelets can reduce failure fracture propagations within the cement [54]. These other additives used together with ferrierite could potentially provide a wide coverage against the many attacks to the cement seen in harsh wellbore environments.

## Figures and Tables

**Table 1:** Oxides present in neat Class-H cement, geothermal zeolite cement and ferrierite by weight percent.

Oxide Formula	Class-H Cement (%)	Commercial Geothermal Zeolite Cement (%)	Ferrierite (%)
Al_2_O_3_	1.47	6.47	8.16
SiO_2_	16.28	29.65	82.43
SO_3_	6.97	9.48	n/a
K_2_O	0.53	1.81	6.05
CaO	68.08	46.27	1.12
TiO_2_	0.23	0.52	0.13
MnO	0.1	0.26	0.01
Fe_X_O_Y_	5.93	5.31	1.81
ZnO	0.14	0.06	n/a
SrO	0.28	0.17	0.04

**Table 2:** Calculated base weights of various components used in the cement mix design for the respective cement formulations. The quantities were scaled up or down in the same proportions depending on the requirements.

Materials Used	Neat Cement (Grams)	5% FER (Grams)	15% FER (Grams)	30% FER (Grams)
Class-H cement	557.80	537.17	500.17	453.34
Water	214.03	208.33	198.11	185.17
Ferrierite	0.00	26.86	75.03	136.00
D-Air 5000	1.40	1.34	1.25	1.13
CFR-3	1.67	1.61	1.50	1.36
Bentonite	11.16	10.74	10.00	9.07

**Table 3:** Summary of mechanical tests for 16.4 ppg neat Class-H, and 5%, 15%, and 30% ferrierite-added Class-H cement formulations.

Sample	Indentation		Triaxial		UCS
	Hardness (GPa)	Elastic (Young’s) Modulus (GPa)	Peak Axial Stress (MPa)	Elastic (Young’s) Modulus (GPa)	Compressive Strength (MPa)
Neat (0% Fer)	0.506	15.72	53.30	9.65	48.55
5% Fer	0.394	14.03	61.13	8.82	68.49
15% Fer	0.319	11.01	62.97	7.72	45.86
30% Fer	0.256	7.88	54.77	11.29	43.57

**Table 4:** Summary of compressive strength and petrophysical tests for 16.4 ppg neat Class-H, and 5%, 15% and 30% ferrierite-added Class-H cement formulations.

Sample Design	UCS	Porosity	Permeability
	Compressive Strength (MPa)	Percentage (%)	Micro-Darcy (μD)
Neat (0% Fer)	48.55	28.82	49.53
5% Fer	68.49	29.75	13.54
15% Fer	45.86	30.29	57.25
30% Fer	43.57	24.62	22.58

## Data Availability

Direct contact to all authors.

## Abbreviations

API	American Petroleum Institute
ASTM	American Society for Testing and Materials
bwoc	By weight of cement
CCS	Confined compressive strength
C_3_A	Tricalcium aluminate
C_2_S	Dicalcium silicate
C_3_S	Tricalcium silicate
C-S-H	Calcium silicate hydrate
CT	Computed tomography
EDS	Energy dispersive spectroscopy
FER	Ferrierite cement
HPHT	High-pressure and high-temperature
MIP	Mercury intrusion porosimetry
P&A	Plugging and abandonment
SEM	Scanning electron microscopy
UCS	Unconfined compressive strength
XRD	X-ray diffraction
XRF	X-ray fluorescence
